# Mitochondrial DNA Maintenance Is Regulated in Human Hepatoma Cells by Glycogen Synthase Kinase 3β and p53 in Response to Tumor Necrosis Factor α

**DOI:** 10.1371/journal.pone.0040879

**Published:** 2012-07-20

**Authors:** Nathalie Vadrot, Sarita Ghanem, Françoise Braut, Laura Gavrilescu, Nathalie Pilard, Abdellah Mansouri, Richard Moreau, Florence Reyl-Desmars

**Affiliations:** 1 INSERM U773, CRB3, Equipe Moreau, Université Paris 7 Denis Diderot, Faculté de Médecine X Bichat, Paris, France; 2 INSERM U773, CRB3, Equipe El-Benna, Université Paris 7 Denis Diderot, Faculté de Médecine X Bichat, Paris, France; University of Medicine and Dentistry of New Jersey, United States of America

## Abstract

During chronic liver inflammation, up-regulated Tumor Necrosis Factor alpha (TNF-α) targets hepatocytes and induces abnormal reactive oxygen species (ROS) production responsible for mitochondrial DNA (mtDNA) alterations. The serine/threonine Glycogen Synthase Kinase 3 beta (GSK3β) plays a pivotal role during inflammation but its involvement in the maintenance of mtDNA remains unknown. The aim of this study was to investigate its involvement in TNF-α induced mtDNA depletion and its interrelationship with p53 a protein known to maintain mtDNA copy numbers. Using quantitative polymerase chain reaction (qPCR) we found that at 30 min in human hepatoma HepG2 cells TNF-α induced 0.55±0.10 mtDNA lesions per 10 Kb and a 52.4±2.8% decrease in mtDNA content dependent on TNF-R1 receptor and ROS production. Both lesions and depletion returned to baseline from 1 to 6 h after TNF-α exposure. Luminol-amplified chemiluminescence (LAC) was used to measure the rapid (10 min) and transient TNF-α induced increase in ROS production (168±15%). A transient 8-oxo-dG level of 1.4±0.3 ng/mg DNA and repair of abasic sites were also measured by ELISA assays. Translocation of p53 to mitochondria was observed by Western Blot and co-immunoprecipitations showed that TNF-α induced p53 binding to GSK3β and mitochondrial transcription factor A (TFAM). In addition, mitochondrial D-loop immunoprecipitation (mtDIP) revealed that TNF-α induced p53 binding to the regulatory D-loop region of mtDNA. The knockdown of p53 by siRNAs, inhibition by the phosphoSer^15^p53 antibody or transfection of human mutant active GSK3βS9A pcDNA3 plasmid inhibited recovery of mtDNA content while blockade of GSK3β activity by SB216763 inhibitor or knockdown by siRNAs suppressed mtDNA depletion. This study is the first to report the involvement of GSK3β in TNF-α induced mtDNA depletion. We suggest that p53 binding to GSK3β, TFAM and D-loop could induce recovery of mtDNA content through mtDNA repair.

## Introduction

In the chronic liver inflammation, the release of pro-inflammatory cytokines such as TNF-α is mainly increased from activated macrophages or monocytes [1]. The major targets of TNF-α are neutrophils, endothelial cells, fibroblasts and hepatocytes [1], [2]. TNF-α is particularly involved in cirrhosis [3], [4]. Cytokine overproduction can lead to hepatopathies and cancers in which mitochondrial dysfunction is a major mechanism [5]–[7]. Permeabilization or rupture of the mitochondrial membrane can occur and provoke liver cell necrosis or apoptosis [1], [2], [7]. Besides these effects, TNF-α induces hepatocyte proliferation through JNK/SAPK activation and survival pathways through NFκB transcription factor can occur [1], [2]. Thus the balance in the liver between cell death and survival, with the latter including proliferation and regeneration, determines cell responses [1], [2].

Up-regulation of TNF-α also generates ROS release [2], [8]. Low level of ROS production plays the role of a second messenger in the different TNF-α signaling pathways [1], [2], [8]. However, abnormal ROS production results in oxidative mtDNA damage, instability and mutations, which can lead to cell transformation and accelerated proliferation [6], [8]. Mitochondria are one of the major sources of ROS in the cell [7]. Because of its close proximity to the respiratory chain, a main source of ROS in cell, of the lack of histone protection and the limited capacity to repair, mtDNA has been suggested to be highly susceptible to oxidative stress [9], [10]. However, the level of base modifications such as adenine and guanine oxidation (8-oxo-dA and 8-oxo-dG) has been estimated as not extensive [11]. In our laboratory, we reported *in vivo* that ROS formation induced in mice livers by alcohol binge or lipopolysaccharides (LPS) are responsible for mtDNA lesions and depletions [12], [13]. In rat isolated hepatocytes, TNF-α also induces ROS production, 8-oxo-dG formation and mtDNA depletion [14]. Oxidative damages can lead to abasic sites, mtDNA strand breaks, deletions and depletions and cause mitochondrial hepatopathies and cancers [5], [6], [9]–[14].

Oxidative stress generated by ROS formation also activates tumor suppressor p53 [15]. Resulting post-translational modifications trigger two p53 subcellular localizations, nuclear and mitochondrial, where this protein has different functions [16]–[17]. During chronic liver inflammation, nuclear p53 stabilized by oxidative stress sensors targets multiple genes involved in growth arrest, apoptosis, DNA repair, senescence or differentiation [18]. Another pool of cytosolic p53 can also translocate to mitochondria and induce transcription-independent mechanisms such as apoptosis, mitochondrial ROS homeostasis, mtDNA base excision repair (mtBER) and copy number maintenance [17], [19]–[21]. We hypothesized that cell exposure to TNF-α could generate ROS and activate mtDNA damage, and that activation of p53 in response to stress could interfere with the damage and provoke mtDNA recovery.

Moreover, during chronic liver inflammation, the serine/threonine kinase GSK3β is a key regulator of cell survival as well as apoptosis [4], [22]. In response to apoptotic stimuli, GSK3β can be present and activated in both, nuclei and mitochondria [23]. The participation of GSK3β in TNF-α induced mtDNA alterations and its interrelationship with p53 are unknown. Its role needs to be investigated since GSK3β may be a therapeutic target.

Thus, the aim of the present study was to assess the involvement of GSK3β in TNF-α induced mtDNA depletion. We also investigated the role of p53 in the regulation of mtDNA content and its interaction with GSK3β. Our results provide new insight into the participation of GSK3β and p53 in mtDNA maintenance. For the first time we report that GSK3β is involved in TNF-α induced mtDNA depletion and that p53 is necessary for the recovery of mtDNA content. We suggest that p53 binding to GSK3β, TFAM and mtDNA regulatory region D-loop could participate in this recovery by stimulating mtDNA repair. These data raise the question of how GSK3β participates in the loss of mtDNA content and how p53 interferes with the inhibition mechanism. Their role in mtDNA damage and repair must be further investigated.

## Results

### TNF-α Did not Induce Apoptosis of HepG2 Cells

**Figure 1:**
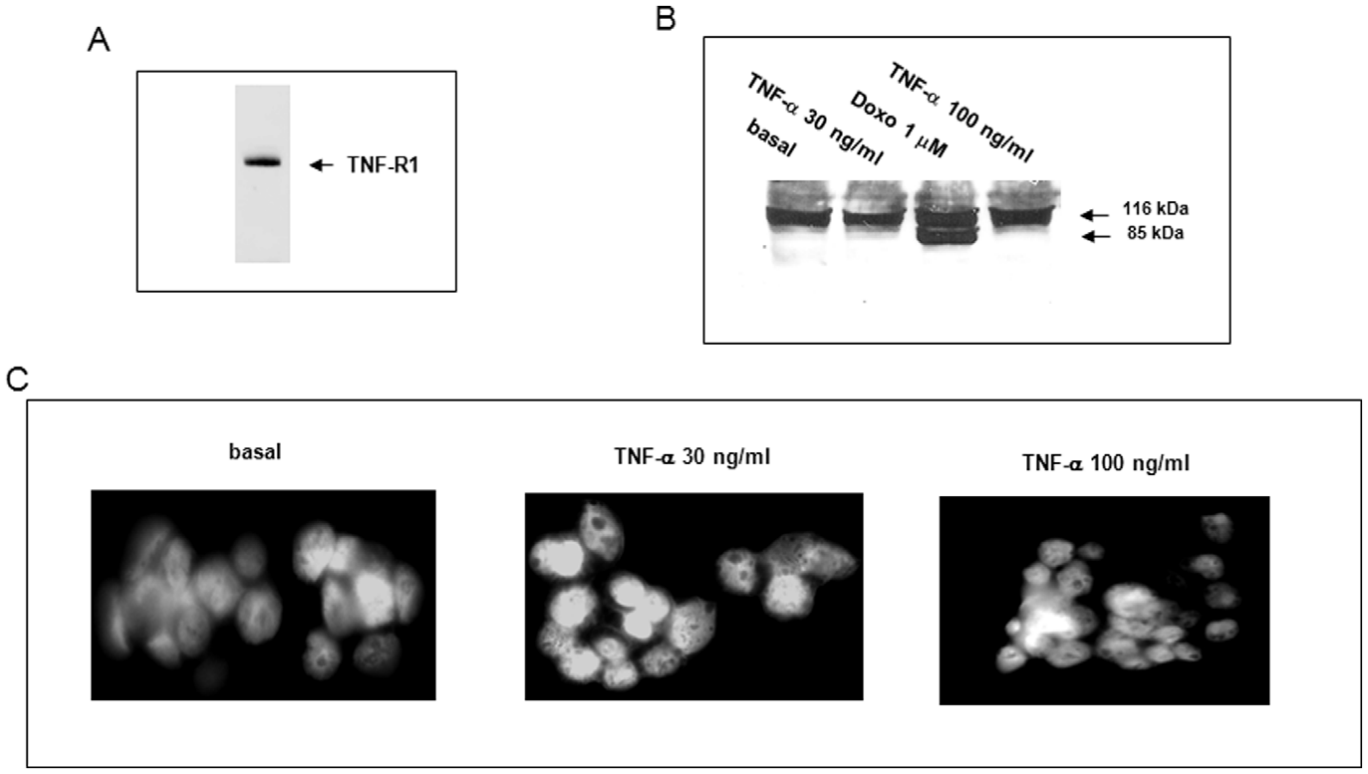
TNF-α did not induce apoptosis of HepG2 cells. (A) Western Blot using the TNF-R1 receptor antibody was performed on cell lysate. (B) PARP cleavage was investigated by Western Blot in cells treated for 18 h with 30 or 100 ng/ml TNF-α or with 1 µM doxorubicin (Doxo) as a positive control. (C) The lack of apoptotic bodies in basal or cells treated with 30 or 100 ng/ml TNF-α has been confirmed by DAPI staining and UV-microscopy.

We first evaluated the effects of TNF-α on HepG2 cell viability. Using flow cytometry and propidium iodide staining we obtained 99.90±0.03% and 99.50±0.05% of viable cells after 18 h treatment with 30 and 100 ng/ml TNF-α respectively. We then used Western Blots to confirm the presence of TNF-R1, a receptor that triggers soluble TNF-α signaling pathways in HepG2 cells [24]. A 55-kDa protein was observed corresponding to the TNF-R1 receptor (Figure 1A). We then examined whether TNF-α induced cell apoptosis. Using Western Blots, we showed that 30 and 100 ng/ml of TNF-α did not induce PARP cleavage at 18 h while as a positive control, 1 µM doxorubicin shown to induce cell apoptosis in our previous paper [25] produced a cleaved fragment of 85 kDa (Figure 1B). The lack of apoptotic bodies observed under UV-microscopy after 30 or 100 ng/ml of TNF-α treatment and DAPI staining is consistent with an absence of apoptosis after 18-h of TNF-α treatment (Figure 1C).

### TNF-α Induced mtDNA Depletion, Lesions and Repair

**Figure 2:**
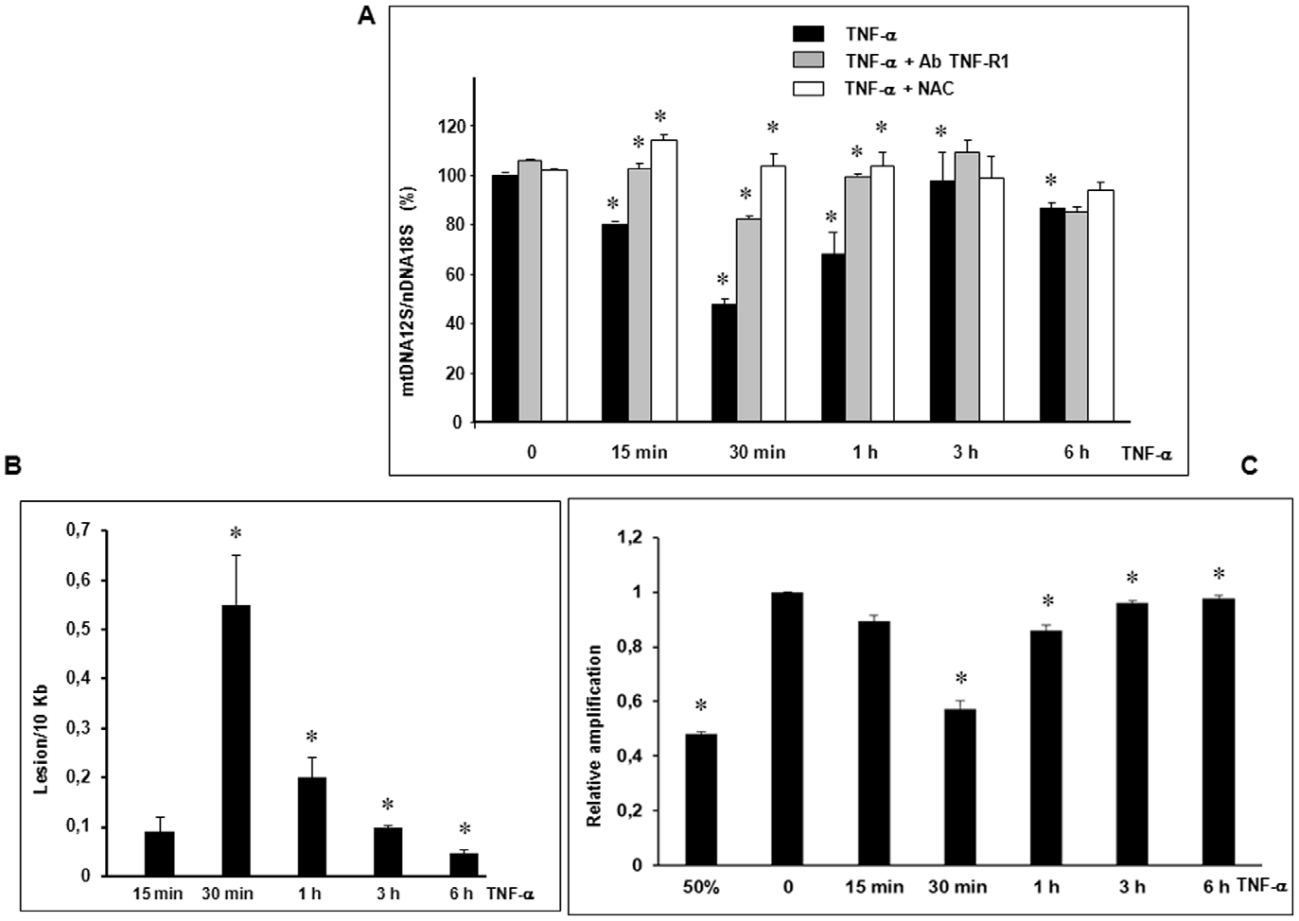
TNF-α induced mtDNA depletion, lesions and repair. (A) Cells were pretreated or not (TNF-α) for 1 h with 1 µg/ml TNF-R1 antibody (TNF-R1 Ab) or with 5 mM NAC. They were then treated for 0 to 6 h with 30 ng/ml TNF-α. To evaluate mtDNA depletion, total genomic DNA was isolated and quantification of mtDNA performed by simultaneous real-time qPCR amplification of fragments encoding mitochondrial 12S rRNA and nuclear 18S rRNA used as a reference gene. Results are expressed in 12S mtDNA over 18S nDNA relative ratio (mean values ± SEM of four independent experiments with four replicates, *p<0.05). (B) mtDNA lesions per 10 Kb were quantified by qPCR amplification of a large fragment (8.9 Kb) from cells treated or not for 15 min-6 h with 30 ng/ml TNF-α and expressed using the Poisson expression [27] (mean values ± SEM of three independent experiments with three replicates, *p<0.05). (C) mtDNA repair activity was measured by calculating relative amplification comparing the values of the treated samples with undamaged control [27], a 50% mtDNA control has been performed (mean values ± SEM of three independent experiments with three replicates, *p<0.05).

We have shown *in vivo* that oxidative stress induced by alcohol or LPS creates mtDNA lesions then mtDNA depletion in the mouse liver [12], [13]. We therefore used real-time qPCR to assess mtDNA content. Nuclear DNA (nDNA) was simultaneously amplified with mtDNA as a control. TNF-α (30 ng/ml) at 30 min significantly decreased mtDNA content by 52.4±2.8% (*p<0.05 *vs* zero-time control) and returned to baseline after 1 to 6 h (Figure 2A). To assess the involvement of the TNF-R1 receptor in the signaling pathway responsible for this depletion, we pretreated cells with the TNF-R1 receptor (CD120a) antibody. The antibody significantly inhibited mtDNA depletion (Figure 2A, *p<0.05 *vs* TNF-α alone). mtDNA alterations are known to be induced by abnormal ROS production which provokes base oxidation [9]–[14]. To assess the involvement of oxidative stress in this depletion, we used the potent antioxidant N-acetylcysteine (NAC), a precursor to glutathione and a ROS scavenger [26]. mtDNA depletion was completely inhibited in the presence of 5 mM NAC (Figure 2A, *p<0.05 *vs* TNF-α alone). These data suggest the presence of TNF-α generated ROS responsible for mtDNA lesions. To evaluate mtDNA lesions we performed qPCR to amplify a large fragment (8.9 Kb) of mtDNA (5999–14841) as described by the Van Houten’s group [27]. This qPCR assay is highly sensitive to low levels of lesions that can block the progression of polymerase along the large fragment and measure the fraction of template molecules that are undammaged [27]. Data analysis revealed the presence of lesions of mtDNA after 30 min of TNF-α exposure estimated at 0.55±0.10 lesions per 10 Kb of mtDNA (*p<0.05 vs 15 min) (Figure 2B). Interestingly, the number of lesions significantly decreased to 0.050±0.005 lesions per 10 Kb, 6 h after TNF-α exposure (*p<0.05 *vs* 30 min) (Figure 2B). We investigated mtDNA repair activity by measuring relative amplification as already described [27]. When mtDNA is damaged, a loss of template amplification is observed and the restoration of the amplification signal represents DNA repair activity [27]. As shown in Figure 2C, a rapid restoration of mtDNA amplification was observed from 30 min to 6 h suggesting the presence of mtDNA repair activity (*p<0.05 *vs* control or *vs* 30 min).

### TNF-α Induced Transient ROS, 8-oxo-dG Production and AP Site Repair

**Figure 3:**
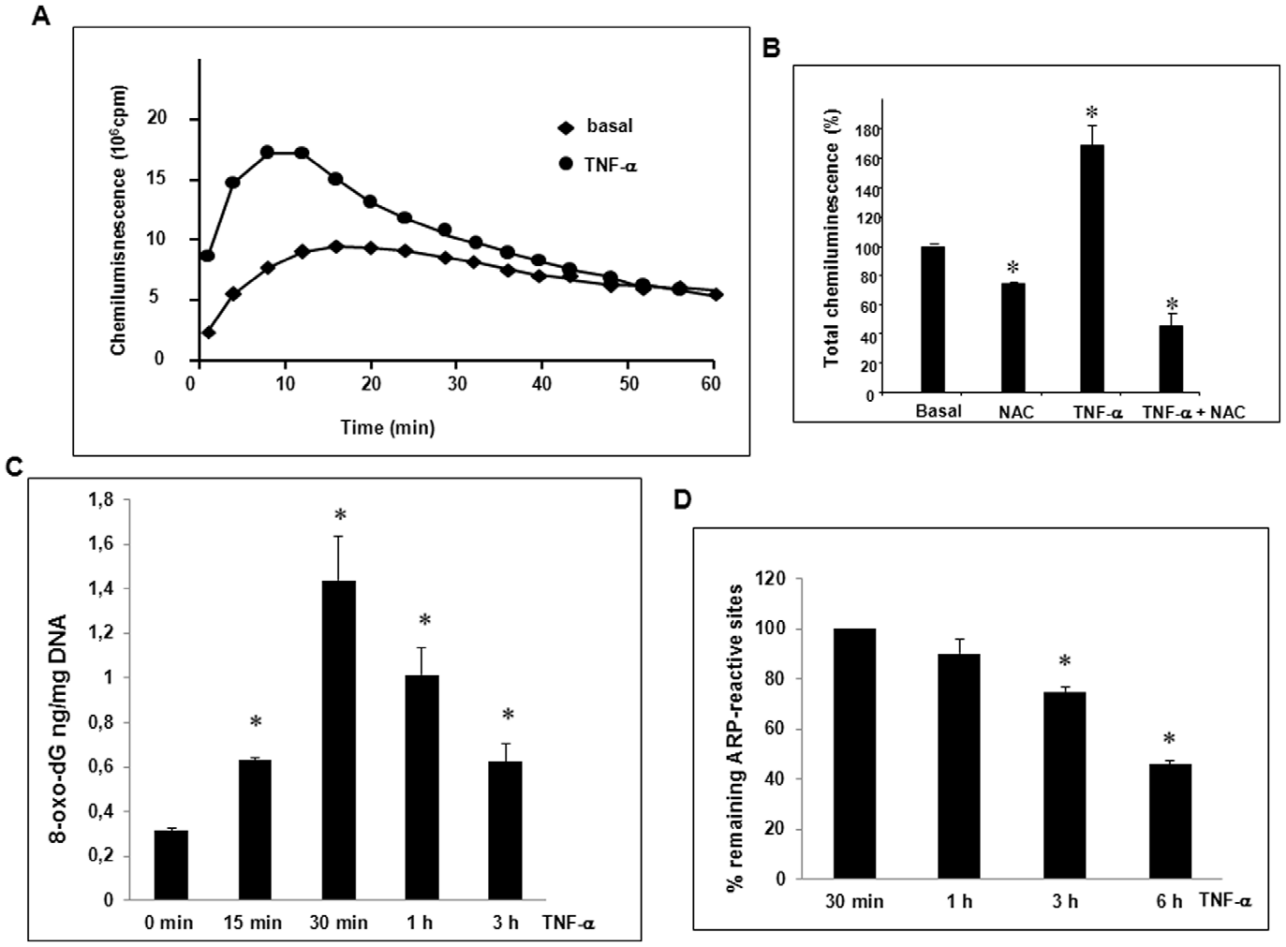
TNF-α induced ROS, 8-oxo-dG production and mtDNA repair. (A) TNF-α induced extra and intracellular ROS were measured over 1 h-period using LAC assay on cell suspension (10^6^ cells in 0.5 ml Hanks buffer) as described in Materials and Methods. One representative experiment of four independent studies is shown. (B) Chemiluminescence is also quantified at the peak in the absence or presence of 5 mM NAC as a percentage of basal value (control) (mean ± SEM for three independent experiments, *p<0.05). (C) TNF-α induced levels of 8-oxo-dG after 15 min to 3 h cell treatment were measured using the OxiSelect™ Oxidative DNA damage ELISA kit (mean values ± SEM of three independent experiments *p<0.05). (D) The decrease of mtDNA remaining ARP-reactive sites created at 30 min TNF-α treatment (100%) was measured from 1 to 6 h using the OxiSelect™ oxidative DNA damage quantitation kit (mean values ± SEM of three independent experiments *p<0.05 *vs* 30 min).

We chose LAC to determine both extracellular released and intracellular retained ROS because luminol is a highly sensitive membrane-permeable molecule [28], [29]. LAC is dependent upon H_2_0_2_ and peroxidases such as cytosolic peroxidases and myeloperoxidases and allows to measure peroxides, anion superoxide and anion hydroxyl levels but cannot distinguish these oxidants from one another [28], [29]. TNF-α generated transient ROS production with a maximum at approximately 10 min corresponding to an increase of 168±15% at the peakcompared to basal cells (*p<0.05 *vs* basal) (Figure 3A). We used NAC [26] to control specificity. Basal and peak TNF-α stimulated ROS productions were decreased by 26.0±0.1% and 72.7±8%, respectively in response to NAC cell pretreatment (*p<0.05 *vs* basal or TNF-α alone) (Figure 3B). Generation of ROS suggested induction of oxidized bases such as 8-oxo-dA and 8-oxo-dG and AP sites [9]–[14]. Results of the ELISA assay showed a level of 8-oxo-dG evaluated to 1.4±0.3 ng/mg DNA at 30 min of TNF-α cell treatment (Figure 3C) close to that observed by Nagakawa and coworkers in rat hepatocytes [14]. A decrease of 8-oxo-dG was observed from 1 to 3 h suggesting mtDNA repair activity. To evaluate mtDNA repair we choose to measure the decrease of the formation of apurinic/apyrimidinic (AP or abasic sites), one specific type of damage among numerous types of oxidative DNA lesions, using an aldehyde reactive probe (ARP) to react specifically with an aldehyde group on the open ring form of AP sites. We measured the decrease of remaining ARP-reactive sites from 30 min of TNF-α treatment (100% of AP sites) to 6 h. A 54.0±1.7% (*p<0.05 *vs* 30 min) decrease of AP sites at 6 h suggested that mtDNA repair occurred within a few hours. This value is close to the 50% restoration of mtDNA content that we observed in figure 2A,C.

### TNF-α Induced p53 Translocation to Mitochondria

**Figure 4:**
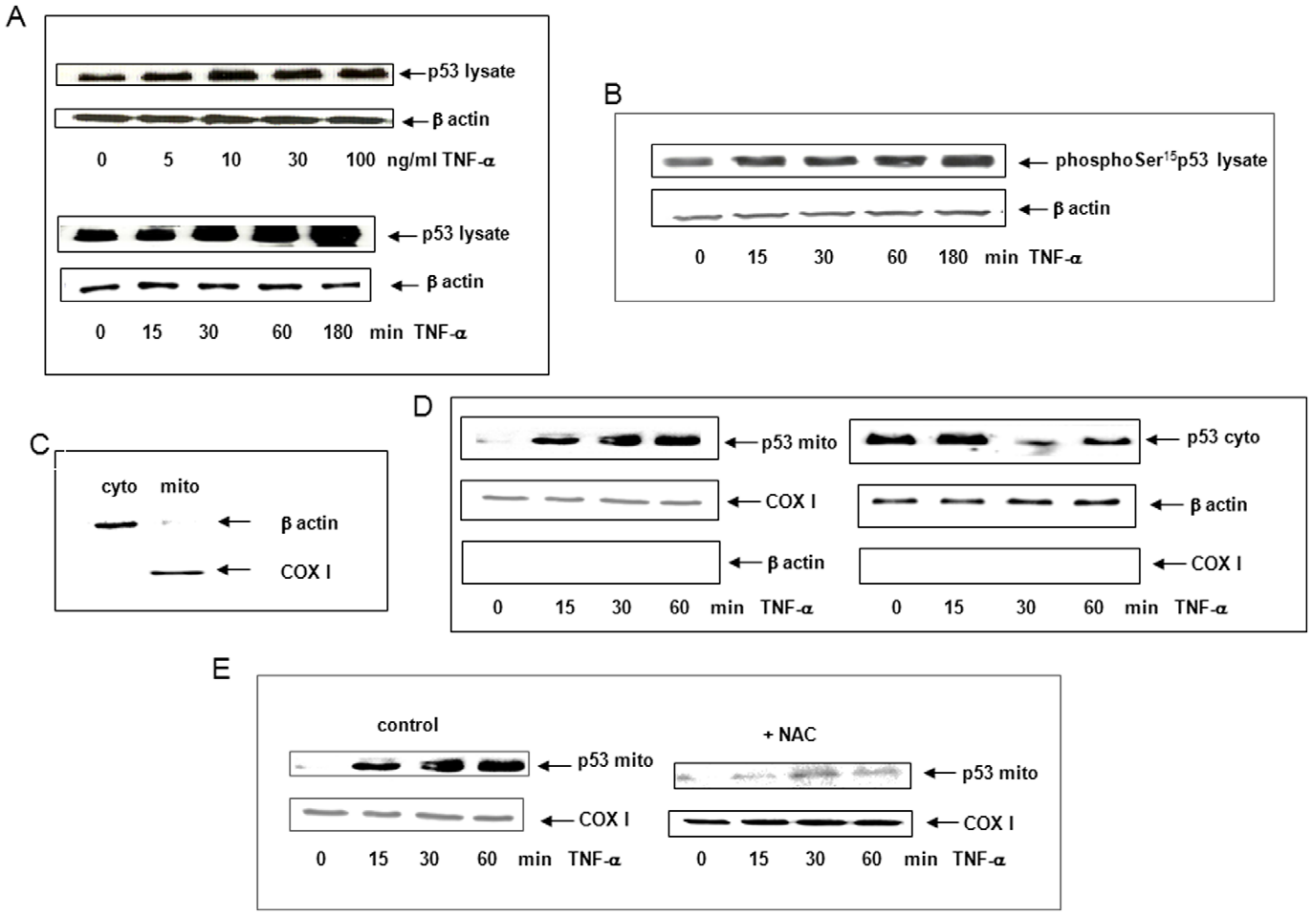
TNF-α induced p53 translocation to mitochondria. (A) Accumulation of p53 was investigated by Western Blot using p53 antibody in lysate from cells treated for 1 h with 5–100 ng/ml TNF-α or with 30 ng/ml TNF-α for 0 to 180 min. β-actin served as a loading control (B) Accumulation of phosphoSer^15^p53 was investigated by Western Blot in lysate from cells treated for 0–180 min with 30 ng/ml TNF-α. β-actin served as a loading control. (C) Mitochondrial (mito) and cytoplasmic fractions (cyto) were isolated as described in Material and Methods and their purities checked by Western Blot using β-actin antibody (cytoplasm) and COXI (mitochondria). (D) The translocation of p53 to mitochondria was investigated by Western Blot using p53 antibody on mitochondrial (mito) or cytoplasmic (cyto) fractions isolated from cells treated or not for 0 to 60 min with 30 ng/ml TNF-α. COXI and β-actin served as loading controls. (E) Cells were pretreated or not (control) for 1 h with 5 mM NAC before exposure to TNF-α. Western Blots were performed using p53 antibody. COXI was used as a loading control.

In the literature, p53 activation has been shown after several hours of TNF-α exposure [18]. In this study, we investigated p53 activation after 15 min-1 h of TNF-α exposure. At one hour, p53 accumulated in response to increasing concentrations of TNF-α with a maximum effect at 30 ng/ml (Figure 4A) and this concentration was used in all other experiments. Western Blots showed that p53 and phosphoSer^15^p53 rapidly accumulated between 30–180 min after TNF-α cell exposure (Figure 4A,B). This accumulation is due to phosphorylation on Ser^15^ which stabilizes p53 and confers its activation [16]. After p53 has stabilized, it can exert mitochondrial transcription-independent functions [17]. We then investigated whether p53 could translocate to mitochondria in response to TNF-α. Mitochondrial and cytoplasmic fractions were isolated and Western Blots were performed using control markers for each compartment to check the purity including cytochrome oxidase I (COXI) and β-actin, respectively. Results showed that β-actin and COXI were not present in mitochondrial or cytoplasmic fractions, respectively, suggesting that fraction separation was good (Figure 4C,D). p53 rapidly accumulated in the mitochondrial fraction between 30 and 60 min after TNF-α cell exposure while p53 expression decreased in the cytoplasm during the same period (Figure 4D) compared to COXI and β-actin used as markers of specificity. To assess whether ROS were involved in p53 translocation to mitochondria, we treated cells with NAC before TNF-α cell exposure. NAC inhibited p53 translocation to mitochondria suggesting that ROS were involved (Figure 4E).

### TNF-α Induced p53 Binding to GSK3β, TFAM and D-loop

**Figure 5:**
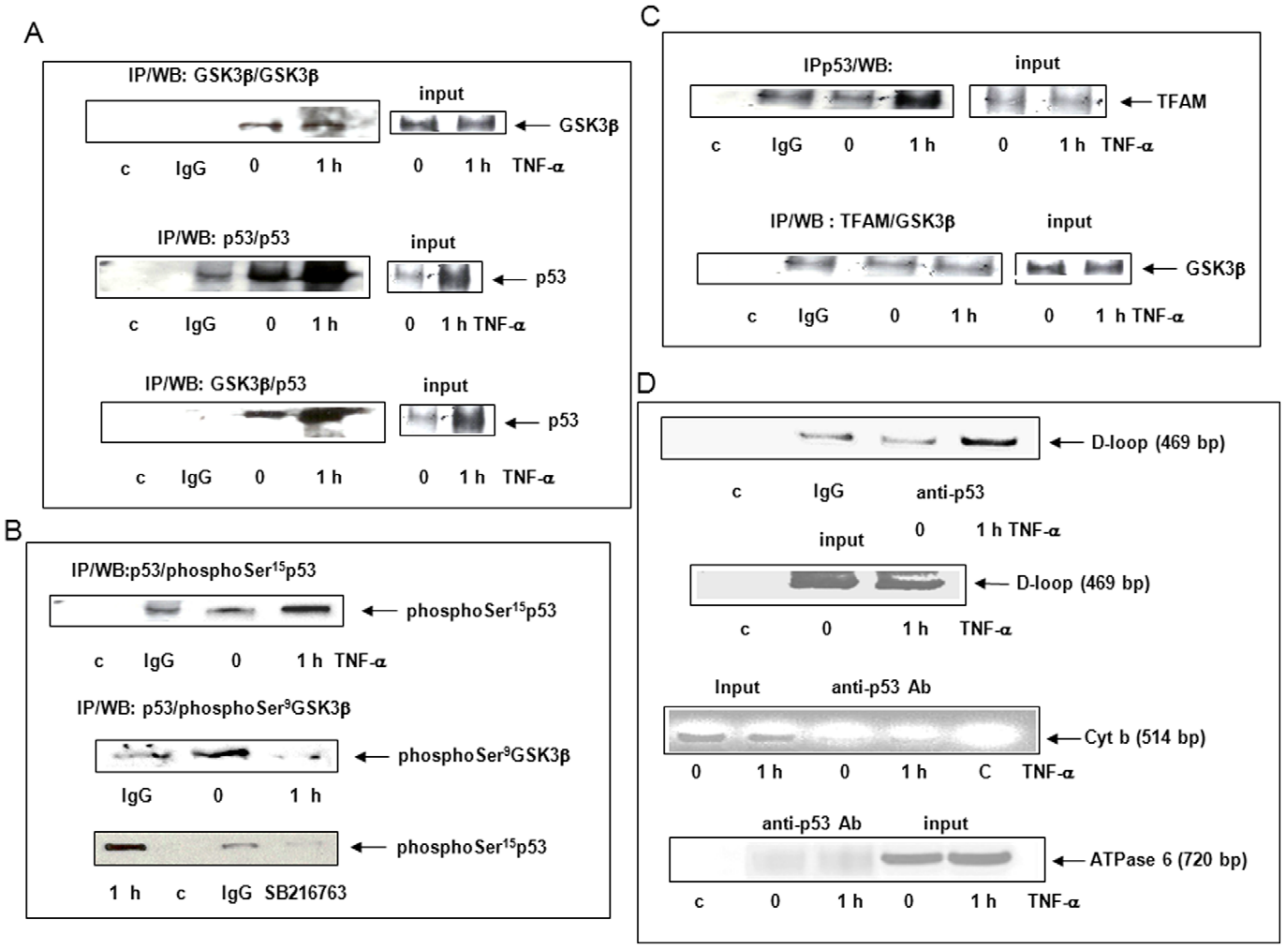
TNF-α induced p53 interaction to GSK3β, TFAM and D-loop. Cells were treated for 0 (zero-time control) or 1 h with 30 ng/ml TNF-α. Mitochondrial fractions were isolated and co-immunoprecipitated or not (input) with GSK3β, p53 (FL-393) or TFAM polyclonal antibodies, IgG or no antibody was used as a control (c). (A) Western Blots were performed using GSK3β or p53 (DO1) antibody. (B) Western Blots were performed using phosphoSer^15^p53 or phosphoSer^9^GSK3β antibody in not pretreated or pretreated cells with GSK3β inhibitor SB216763. (C) Western Blots were performed using TFAM or GSK3β antibody. (D) The mtDIP assay was performed with cross-linked DNA prepared from treated cells for 0 (zero-time control) or 1 h with 30 ng/ml TNF-α. Immunoprecipitates were performed without (control, c) or with IgG used as a control or with p53 antibody. PCR were realized on immunoprecipitates or inputs using a primer pair covering D-loop, cytochrome b (cyt b) or ATPase 6 (Figure 5D).

To further identify the mechanisms of mitochondrial p53 induced by TNF-α, we investigated putative partners, in particular serine/threonine kinase GSK3β, a protein localized and activated in nuclei and mitochondria and known to play a pivotal role in chronic liver inflammation [4], [22], [23]. Co-immunoprecipitation assays were performed to estimate GSK3β expression in the mitochondrial fraction. Results showed that GSK3β was constitutively present in the mitochondria while p53 expression and interaction with GSK3β were enhanced 1 h after TNF-α cell exposure (Figure 5A). We evaluated phosphorylation states of mitochondrial p53 and GSK3β (Figure 5B). PhosphoSer^15^p53 was induced whereas Ser^9^GSK3β present at zero-time was dephosphorylated after 1 h of TNF-α cell exposure. Cell pretreatment for 1 h with 80 µM SB216763, an inhibitor of GSK3β activity [30] inhibited Ser^15^p53 phosphorylation (Figure 5B). We also investigated the interaction of p53 and TFAM, a transcription factor involved in mtDNA transcription/replication machinery, repair and nucleoid structure [31]–[33]. Co-immunoprecipitation assays revealed that p53 can interact with TFAM after 1 h of TNF-α cell exposure while no binding was observed between TFAM and GSK3β (Figure 5C). To investigate a putative binding of p53 to the D-loop of mtDNA, a non-coding regulatory region in which we measured lesions, we performed the mtDIP assay derived from ChIP [34]. A 469 bp D-loop fragment was amplified on PCR, with the p53 antibody after 1 h of TNF-α cell exposure, while no specific signal was obtained without the antibody or when IgG was used for immunoprecipitation (Figure 5D). No PCR products were amplified when primers corresponding to cytochrome b or ATPase 6 were used as controls (Figure 5D).

### The p53 Inhibition by siRNAs and PhosphoSer^15^p53 Antibody Prevented the Reversion of mtDNA Depletion

**Figure 6:**
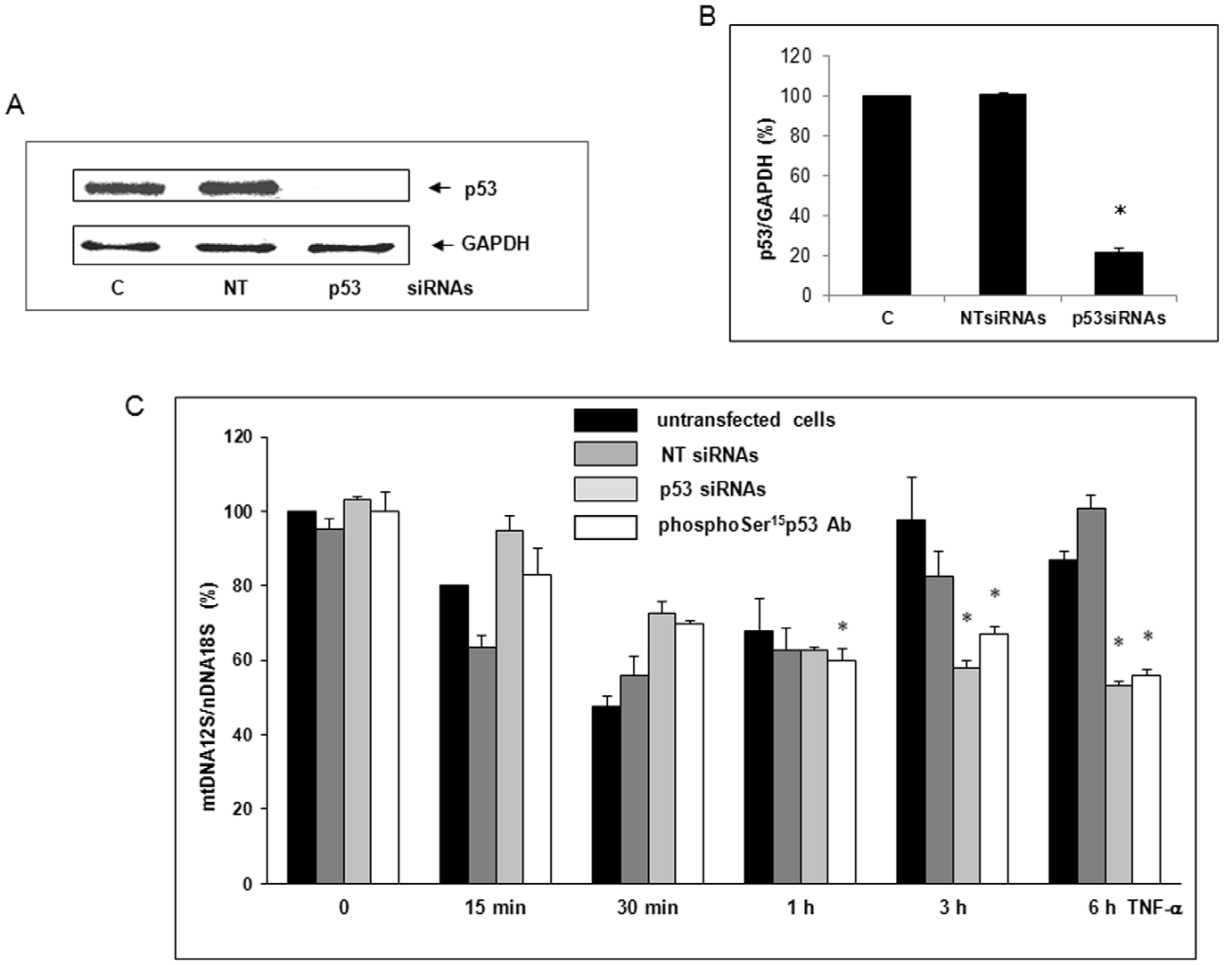
p53 inhibition by siRNAs and phosphoSer^15^p53 antibody prevented the reversion of mtDNA depletion. (A) All siRNA transfections were performed with 12.5 nM siRNAs directed against p53 mRNA (p53 siRNAs) or non-targeting siRNAs (NT) and DharmaFECT4® transfection reagent used as a control (c). To check siRNA transfection efficiency, Western Blots were performed at 48 h using p53 or GAPDH (loading control) antibody. (B) The knockdown of p53 expression by siRNAs relative to GAPDH was quantified using the Bio1D software (mean values ± SEM of three experiments *p<0.05) (C) DNA was isolated from untransfected cells or transfected with NT siRNAs or with p53 siRNAs. Cells were also permeabilized with 0.1% Triton X100 and pretreated for 1 h with 1 µg/ml phosphoSer^15^p53 antibody (phosphoSer^15^Ab). Cells were then treated for 0 to 6 h with 30 ng/ml TNF-α. The quantification of mtDNA content was performed by simultaneous real-time qPCR amplification of fragments encoding mitochondrial 12S rRNA and nuclear 18S rRNA serving as a reference gene. Results are expressed in mtDNA over nDNA relative ratio (mean values ± SEM of three independent experiments with five replicates, *p<0.05).

p53 is known to protect the mitochondrial genome by stimulating base excision repair (BER) and/or replication allowing maintenance of mtDNA copy number [19]–[21]. To evaluate whether p53 was involved in the recovery of mtDNA content observed in Figure 2 A, siRNAs known to knockdown p53 expression were used [35]. To check siRNA transfection efficiency, we performed Western Blots at 48 h. p53 expression was decreased by 78.3±2.5% (*p<0.05 *vs* control) while Dharmafect4® alone (C) or non-targeting siRNAs (NT) used as controls had no effect on p53 expression (Figure 6A,B). Interestingly, p53 knockdown by siRNAs impaired the reversion of mtDNA depletion observed from 1–6 h of TNF-α cell exposure (*p<0.05 *vs* untransfected cells) while no significant effect was observed with NT siRNAs (Figure 6C). At 6 h, mtDNA content was evaluated at 46.7±1% (*p<0.05, *vs* zero-time control), a value close to baseline. In addition, pretreatment of the permeabilized cell with phosphoSer^15^p53 antibody prevented the reversion of mtDNA depletion (Figure 6C). These data suggested that p53 could be involved in the recovery of mtDNA content.

### SB216763 or GSK3β siRNAs Inhibited mtDNA Depletion Whereas GSK3βS9A Suppressed the Reversion

**Figure 7:**
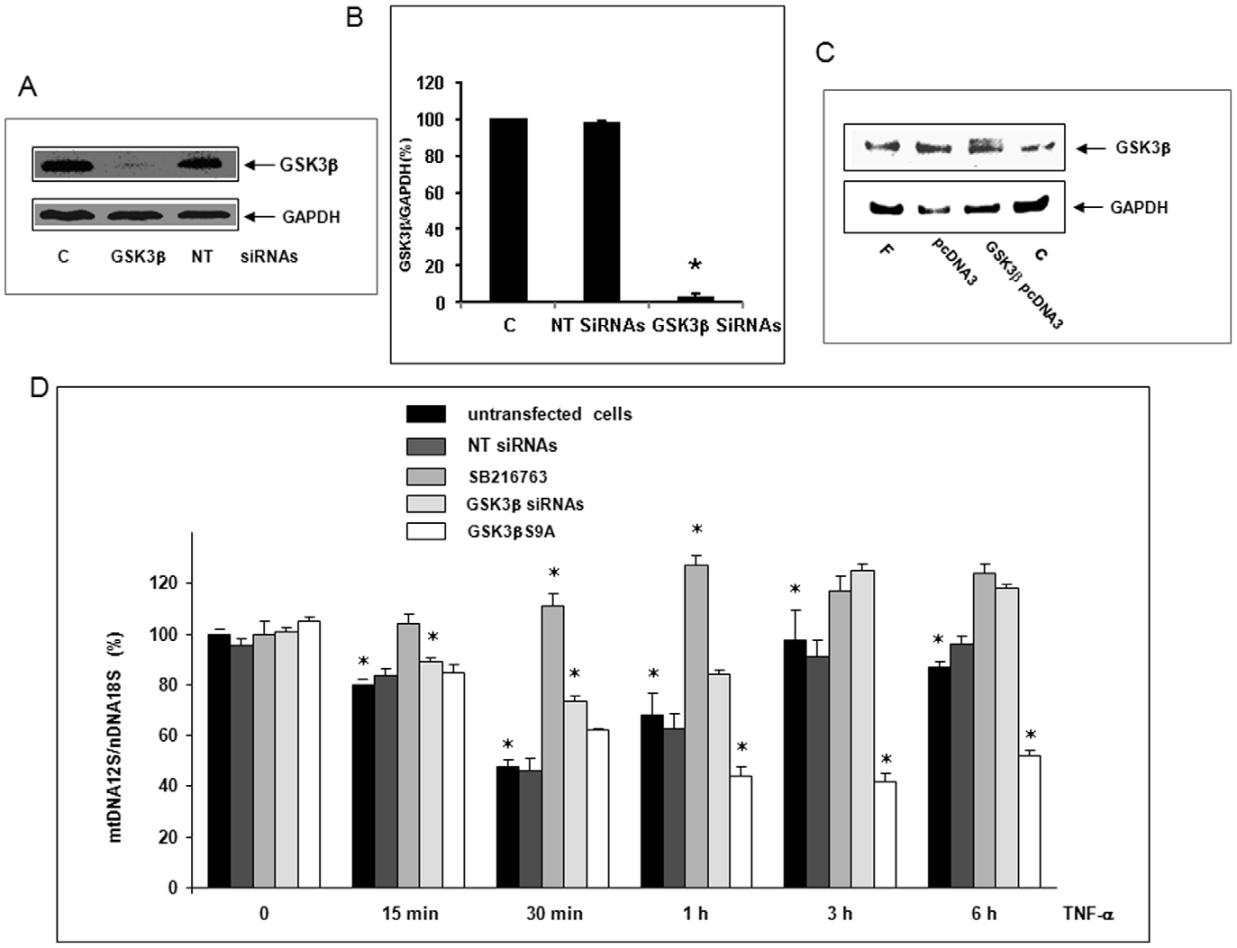
SB216763 or GSK3β siRNAs inhibited mtDNA depletion whereas GSK3βS9A suppressed the reversion. (A) All siRNA transfections were performed or not (control, C) with 12.5 nM siRNAs directed against GSK3β mRNA (GSK3β siRNAs) or non-targeting (NT) siRNAs in DharmaFECT4® transfection reagent. To check SiRNA transfection efficiency, Western Blots were performed at 48 h with GSK3β or GAPDH (loading control) antibody. (B) Inhibition of GSK3β expression by siRNAs relative to GAPDH was quantified using the Bio1D software (mean values ± SEM of three experiments *p<0.05) (C) Cells were transfected or not (control C or Fugene HD® alone, F) with the mutant GSK3βS9A pcDNA3 plasmid or the empty plasmid (pcDNA3) using Fugene HD® (F). After 72 h transfection, the expression of recombinant GSK3βS9A protein was checked by Western Blot using GSK3β or GAPDH (loading control) antibody. (D) Cells were treated or not with SB216763 or transfected or not (untransfected cells) with NT siRNAs or GSK3β siRNAs or GSK3βS9A pcDNA3 plasmid. Then, they were treated for 0 (zero-time control) to 6 h with 30 ng/ml TNF-α. Total DNA was isolated and the quantification of mtDNA content performed by real-time qPCR co-amplification of fragments encoding mitochondrial 12S rRNA and nuclear 18S rRNA as a gene reference. Results are expressed in mtDNA over nDNA relative ratio (mean values ± SEM of three independent experiments with five replicates, *p<0.05).

To investigate the involvement of GSK3β in mtDNA depletion, we pretreated cells with the GSK3β inhibitor SB216763 [30]. Cells were also transfected with GSK3β siRNAs known to inhibit its expression [36]. To confirm the involvement of GSK3β in mtDNA depletion, cells were also transfected with a mutant GSK3βS9A in which Ser^9^ is replaced by Ala^9^, impairing phosphorylation and then inhibition of GSK3β activity [37]. siRNA transfection efficiency was checked at 48 h by Western Blot. GSK3β siRNAs inhibited protein expression by 96.8±1.5% (*p<0.05 *vs* control) while Dharmafect4® alone (c) or NT siRNAs as controls had no effect (Figure 7A,B). We checked the presence of recombinant GSK3βS9A protein by Western Blot after 72 h transfection (Figure 7C). In cells pretreated with SB216763 or transfected with GSK3β siRNAs, mtDNA depletion was inhibited and reversion enhanced (*p<0.05 *vs* non-transfected cells) (Figure 7 D). In cells expressing GSK3βS9A, the reversion of mtDNA depletion was completely inhibited to a value close to baseline (46.3±1.2% of remaining mtDNA at 6 h, *p<0.05 *vs* non-transfected cells)**.** These data suggest the involvement of GSK3β in mtDNA depletion.

## Discussion

The aim of this study was to investigate whether GSK3β is involved in the loss of TNF-α induced mtDNA content and counteracted by p53. For the first time we found that GSK3β was involved in mtDNA depletion in response to TNF-α in hepatoma cells. These data suggest that TNF-α induces mtDNA alterations and that GSK3β could be involved in the activation of these alterations or in the inhibition of mtDNA repair. This mechanism remains unknown, although recently, one study showed that in hippocampal neuron nuclear DNA, GSK3β inhibits non-homologous end-joining-mediated repair of double strand break induced by irradiation [38]. Further experimental studies are necessary to identify the putative inhibitory effects of GSK3β in BER the main pathway to repair mtDNA [9]–[11], [19]–[21].

According to the NAC antioxidant effect, the induction of mtDNA depletion could appear in response to ROS production. Production of ROS is also transient. This could reflect a burst or flash then release of ROS as reported in the literature [39]. The TNF-α induced 8-oxo-dG and AP sites production that we observed also supports the involvement of ROS in mtDNA damage as a cause of mtDNA depletion [11]–[14]. This depletion reflects the loss of undamaged template due to degradation of damaged mtDNA molecules being not amplified by qPCR [27], [40].

Experiments using SiRNA knockdown have shown that the p53 protein was necessary for the reversion of depletion. Repletion at 1 h is probably not due to the transcription of nuclear genes related to the generation of ROS but is probably linked to mitochondrial transcription-independent mechanisms [17]. The repletion of mtDNA that we observed could result in the repair of damaged mtDNA. As the damage is repaired, amplification is restored and reflects the kinetic of DNA repair [27]. Relative amplification of mtDNA and decrease of ARP-reactive sites appearing from 1 to 6 h suggested a rapid and efficient mtDNA repair of this type of oxidative damage as suggested in the literature [10]. Our previous *in vivo* studies and some *in vitro* studies in the literature have shown a rapid turnover of mtDNA in hepatocytes to maintain the integrity of information [12], [13], [41], [42]. Knockout of p53 results in a 50% reduction in mtDNA copy number in mouse neonatal fibroblasts [19]. Down-regulation of packaging factor TFAM and p53-regulated subunit of ribonucleotide reductase (p53R2), two proteins involved in mtDNA maintenance are also observed in the absence of p53 [19]. We also hypothesized the presence of mtDNA repair since p53 has been shown to be involved in mtBER in the case of mtDNA oxidative damage [3], [44]. Base excision repair (BER) is the main pathway for the repair of 8-oxo-dG [9]–[11] involving the mitochondrial bifunctional 8-oxodG DNA glycosylase/apurinic DNA lyase (OGG1) [11]. In p53^−/−^ H1299 and HCT116 human colorectal cancer cells, decreased excision of misincorporated nucleotides and exonuclease activity were observed [43], [44]. p53 can also participate to mtBER by interacting with polymerase γ, stimulating nucleotide incorporation increasing glycosylase step and exonuclease activity of DNA polymerase γ [34].

In the present study, p53 also interact with TFAM as described in the literature [45]. TFAM is a member of the family of HMG-box proteins involved in replication and transcription through its interaction with the D-loop region in particular in case of oxidative damage [31]–[32], [46], [47]. TFAM has also been shown to maintain mtDNA content as a main component of the assembly of multiple DNA molecules into nucleoid-like structures inducing mtDNA packaging [33]. According to a recent study, TFAM inhibits mtBER enzyme activity [48]. The interaction of p53 with TFAM could prevent inhibition of mtDNA repair by preventing its interaction with the non-coding regulatory region D-loop [48]. Therefore, we suggest that the binding of p53 to TFAM, observed one-hour after TNF-α cell exposure could participate in the recovery of mtDNA content. We will further investigate the role of TFAM in mtDNA depletion by knockdown experiments. Besides binding to TFAM, p53 binds to the D-loop, a regulatory region containing transcription and replication origins [9]. This also suggests that p53 may play a role in mtDNA replication. A role in mtDNA repair can be also suggested since our data are supported by results in the literature showing that in response to oxidative stress, p53 can bind to single-stranded regions of the D-loop, enhancing its intrinsic exonuclease activity especially for the excision of 8-oxo-dG [19], [43], [44]. We will study the presence of oxidative damage in D-loop region bound to p53. In contrast, TFAM does not seem to be a direct substrate of GSK3β. We suggest that the interaction of p53 and GSK3β could induce binding to TFAM followed by concomitant release of GSK3β. We will examine the direct role of p53 in the binding of TFAM and D-loop in further studies.

All of our results support early p53 translocation to mitochondria. Oxidative stress generated by ROS induces post-translational modifications of p53 [15]–[17]. At one-hour, a pool of phosphoSer^15^p53 accumulates in mitochondria in response to TNF-α. This phosphorylation activates p53 by inhibiting its binding to hdm2, a key regulator that functions as an ubiquitin ligase promoting p53 degradation [16]. In resting HepG2 cells, mitochondrial phosphorylated Ser^9^GSK3β was found to be expressed in a constitutive manner in mitochondria. GSK3β activity is known to be inhibited through Ser^9^ phosphorylation which, occurs by many upstream kinases, including Akt/PKB kinase [49]. In normal cells, Ser^9^GSK3β is dephosphorylated whereas in some hepatoma cells, GSK3β is constitutively phosphorylated on Ser^9^ by an up-regulated PI-3kinase/Akt signaling pathway [49], [50]. Akt is rapidly translocated to mitochondria when the PI-3kinase-signaling pathway is activated [50]. This supports the fact that mitochondrial GSK3β was Ser^9^phosphorylated in resting HepG2 cells since Akt is activated in these cells. Further experiments are needed to clarify the mechanisms by which TNF-α induces Ser^9^GSK3β dephosphorylation and activation of GSK3β. Interestingly, expression of the recombinant active GSK3βS9A mutant protein in which Ser^9^ phosphorylation is impaired increases mtDNA depletion. This is consistent with the inhibition of mtDNA depletion when GSK3β is knocked down. These data confirm that GSK3β is activated by TNF-α by dephosphorylation and participates in mtDNA depletion. In addition, we have shown that p53 Ser^15^ phosphorylation is necessary for repletion and that p53 binds to GSK3β. The results of the present study suggest that activated p53 interacts with GSK3β, inhibits its functions and then prevent mtDNA depletion.

In conclusion, during the first minutes of TNF-α cell exposure, ROS production, activation of GSK3β activity, mtDNA lesions and depletion are induced in HepG2 cells. We suggest that reversion of mtDNA depletion occurs in response to p53 translocation to mitochondria and binding to GSK3β, TFAM and D-loop. For the first time we suggest that GSK3β, a serine/kinase activated in chronic liver inflammation may be a key factor in mtDNA damage and that p53 could counteract its activity for maintaining mtDNA content.

## Materials and Methods

### Cell Culture, Treatments with TNF-α and Reagents

Human HepG2 cell line derived from hepatoblastoma was purchased from ATCC (Rockville, MD, USA). HepG2 cells are a model commonly used for studying wild type p53 [51], [52] and GSK3β signaling pathways [49]. Cells were cultured at 37°C in DMEM (Invitrogen, Cergy Pontoise, France) supplemented with 10% heat-inactivated FBS Clone (PAA, Les Mureaux, France), 100 IU/ml penicillin/100 µg/ml streptomycin and 10 µg/ml gentamycin (Invitrogen). In all experiments, TNF-α at 30 ng/ml (R&D System, Lille, France) or IFN-γ (R&D System) at 100 ng/ml or doxorubicin (Sigma Aldrich, Lyon, France) at 1 µM was added when necessary to culture medium for 0 to 18 h. This dose-range commonly used in the literature [14] provided us with maximal effects to induce p53 translocation in HepG2 cells. When necessary, 5 mM antioxidant NAC, 80 µM GSK3β inhibitor arylindole-maleimide SB216763 (Sigma Aldrich), 1 µg/ml TNF-R1 receptor antibody (CD120a) (Millipore, Molsheim, France) or phosphoSer^15^p53 antibody (Cell Signaling Technology, Ozyme, St Quentin en Yvelines, France) were added for 1 h before cell exposure to TNF-α.

### Cell Transfections

All siRNA transfections were performed using a pool of four siRNAs targeting different regions of p53 [35] or GSK3β [36] mRNA (ON-TARGET*plus*® SMARTpool, Thermo Fisher Scientific, Lafayette, CO, USA). A non-targeting (NT) pool of four different siRNAs was used as a negative control (ON-TARGET*plus*® SMARTpool). Cells grown in antibiotic-free complete medium were transfected in Opti-MEM (Invitrogen) using DharmaFECT4® transfection reagent with a final concentration of 12.5 nM siRNAs. All the experiments were performed 48 h after transfection. On the other hand, human GSK3βS9A pcDNA3 mutant plasmid provided from Addgene (Cambridge, MA, USA) was transfected using FuGENE®HD (Promega, Madison,WI, USA) in antibiotic-free complete Opti-MEM. All experiments were performed 72 h after transfection.

### Subcellular Fraction Isolation

To assess expression of p53, GAPDH or GSK3β by Western Blot in cells that were transfected or not and treated or not, lysates of total protein were prepared as already described in the presence of 25 mM Hepes, pH 7, 0.1% Nonidet P40 and a protease inhibitor cocktail (Sigma Aldrich) [25], [51], [52]. Mitochondrial and cytoplasmic fractions were isolated as already described by successive centrifugations using 10 mM Tris HCl, pH 7,5, 10 mM NaCl, 1.5 mM MgCl_2_, 1 mM EDTA, 70 mM sucrose, 210 mM mannitol and a protease inhibitor cocktail (Sigma Aldrich) [52]. Purity of mitochondrial and cytoplasmic compartments was checked by Western Blots using cytochrome c oxidase (COXI) and β-actin antibodies, respectively, as described below. For PARP cleavage analysis, nuclear fractions were extracted as already described in our previous papers [25], [51], [52].

### Mitochondrial D-loop Immunoprecipitation (mtDIP)

The mtDIP assay derived from the chromatin immunoprecipitation (ChIP) was performed according to a previously published modified method [34]. Briefly, mitochondrial fractions isolated from cells treated or not with TNF-α for 1 h were incubated at room temperature with 1% formaldehyde. The cross-linking reaction was stopped by incubation for 10 min with 125 mM glycine followed by two sonications at 30 s intervals and incubated for 10 min in an ice-bath. Samples were then centrifuged at 5000 xg for 10 min. One-tenth supernatant was used as input, a control for PCR. The remaining supernatant was submitted to immunoprecipitation without (control) or with polyclonal antibody against p53 (Santa Cruz, Tebu-Bio, Le Perray en Yvelines, France) or non-specific IgG (Sigma Aldrich) used as a control and protein G-conjugated agarose beads (Tebu-Bio, Le Perray en Yvelines, France). Both input and immunoprecipitated samples were incubated in the presence of elution buffer (1% SDS, 0.1 M Na_2_CO_3_) at 65°C for 15 min and centrifuged at 2000 xg for 5 min. After cross-linking, reversion and treatment with 0.1 mg/ml proteinase K (Invitrogen), DNA was extracted with the Extract II kit Macherey Nagel using the NTB buffer (Macherey Nagel, Hoerdt, France). PCR was performed using a pair of primers specific for D-loop (mt15–mt484), cytochrome b (mt15260–mt15774) or ATPase 6 (mt8539–mt9059) [53] in the GeneAmp PCR System thermocycler according to the following cycling protocol: 94°C for 3 min, 35 cycles, 46°C for 3 min, and 72°C for 1 min with a final extension of 7 min at 72°C. Samples were loaded into Syber® Safe DNA (Invitrogen) stained 1% agarose gel and visualized under a UV transilluminator system (Quantum, Vilber Lourmat, Torcy, France).

### Measurement of mtDNA Depletion, Lesions and Repair by qPCR

To assess mtDNA depletion and lesions, genomic DNA was isolated using phenol/chloroform/isoamylalcohol [34] or QIA amp® DNA kit (Qiagen, Courtabœuf, France) from HepG2 cells transfected or not cells and treated or not with TNF-α. mtDNA quantifications were performed from serial dilutions of DNA by real-time qPCR amplification on a Light Cycler LC430 (Roche Applied Sciences, Meylan, France). Amplifications were monitored and analyzed by measuring the intercalation of the fluorescent dye (Fast Start DNA Master plus SYBR Green1 kit) to double-stranded DNA (Roche Applied Sciences). mtDNA copy number was evaluated by co-amplifying a DNA fragment encoding mitochondrial 12S rRNA and a DNA fragment encoding nuclear 18S rRNA as gene reference (table 1). qPCR amplifications in the absence of primers or DNA were performed as a control. All data were calculated using Light Cycler LC430 software. Since mtDNA lesions blocking replication are more likely to be present on a large mtDNA region than on a short fragment, we performed qPCR amplifications of a long (8,9 Kb) and small (221 bp) fragment (table 1) [27] using 20 ng total DNA isolated from cells that were treated or not with TNF-α. qPCR amplifications were performed in the absence of primers or DNA as a control using a PCR express HYBAID thermocycler. The lesions were estimated according to the Poisson expression as already described [27].

**Table 1:** Primer sequence used for qPCR.

Name	Forward	Reverse
12S	TAGCCCTAAACCTCAACAGT	TGCGCTTACTTTGTAGCCTTCAT
18 S	CCCTGCCCTTTGTACACACC	GATCCGAGGGCCTCACTA
Large mt	TCTAAGCCTCCTTATTCGAGCCGA	TTTCATCATCATGCGGAGATGTTGGATGG
Small mt	CCCCACAAACCCCATTACTAAACCA	TTTCATCATGCGGAGATGTTGGATGG
D-loop	CACCCTATTAACCACTCACG	TGAGATTAGTAGTATGGGAG
GAPDH	ACCCAGAAGACTGTGGATGG	TTCAGCTCAGGGATGACCTT

### Measurement of ROS, 8-oxo-dG Production and mtDNA Repair

LAC was used to measure extra and intracellular ROS productions as described previously [28], [29]. Briefly, after trypsination, cell suspension (10^6^ cells in 0.5 ml Hank’s balanced salt solution) was incubated at 37°C in the thermostated chamber of the luminometer (Berthold-Biolumat LB937) and allowed to stabilize. After a baseline reading, cells were incubated with 10 µM luminol (Sigma Aldrich) and 5 IU/ml horseradish peroxidase (Sigma Aldrich), then stimulated or not with TNF-α and changes in chemiluminescence were recorded over a 1 h period. When necessary, cells were pretreated for 1 h with 5****mM NAC. We also evaluate levels of 8-oxo-dG known to be created by ROS [10], [11], [14] using OxiSelect™ Oxidative DNA damage ELISA kit. mtDNA repair of AP sites was evaluated by measuring remaining ARP-reactive sites using OxiSelect™ oxidative DNA damage quantitation kit (Cell BIOLABS INC, Euromedex, Souffelweyersheim, France). For these purposes DNA was isolated from mitochondrial pellets prepared and checked as described above using QIAamp® DNA kit for genomic and mitochondrial DNA purification (Qiagen, Courtabœuf, France). The purity of isolated mtDNA was determined by PCR using a pair of primers specific for D-loop [53] (table 1) and for GAPDH (table 1) to check the absence of nDNA contamination. PCR were performed in a Perkin Elmer GeneAmp PCR System thermocycler. The cycling protocol for D-loop was as follows: initial denaturation 94°C for 3 min, 35 cycles, 94°C for 1 min, 46°C for 3 min, 72°C for 1 min with a final extension of 7 min at 72°C. The protocol for GAPDH was as follows: initial denaturation 95°C for 3 min, 30 cycles, 94°C for 1 min, 58°C for 1 min, 72°C for 1 min with a final extension of 7 min at 72°C. Samples were loaded into Syber® Safe DNA (Invitrogen) stained 1% agarose gel and visualized under a UV transilluminator system (Quantum, Vilber Lourmat, Torcy, France).

### Co-immunoprecipitations and Western Blots

Co-immunoprecipitations were performed using polyclonal FL-393 antibody against p53 (Santa Cruz Biotechnology), GSK3β (Santa Cruz Biotechnology) or TFAM (Santa Cruz Biotechnology) and agarose protein A/G Plus beads (Santa Cruz) as described in a previous paper [52]. For Western Blots, samples were loaded onto 10–15% SDS-PAGE and separated proteins were electrotransferred for 7 min onto nitrocellulose membrane using the iblot™ System from Invitrogen. Western blots were performed using primary monoclonal antibodies against p53 (DO-1, Santa Cruz Biotechnology), phosphoSer^15^p53 (Cell Signaling Technology), GSK3β (Santa Cruz Biotechnology), phosphoSer^9^GSK3β and TNF-R1 receptor (CD120a) (Millipore, Molsheim, France), TFAM, β-actin and COXI (Santa Cruz Biotechnology) and GAPDH (Sigma Aldrich) used as a control. Chemiluminescence was visualized using the Pierce ECL Western Blotting Substrate (Thermo Fisher Scientific, Brebières, France) under a Fusion Fx7 camera and when necessary, quantification was performed using the Bio1D software (Vilber Lourmat, Torcy, France).

### Analysis of Apoptosis

The presence of chromatin condensation was analysed under UV-light microscopy after cell staining with di-amino-phenyl-indole (DAPI) (Sigma Aldrich) as already described [25]. PARP cleavage was studied by Western Blot on nuclear extracts using a monoclonal anti-PARP antibody (PharMingen, Lexington, KY) as already described [25], [52]. Cell viability was checked by flow cytometry after 15 min cell incubation with 1 µg/ml propidium iodide.

### Statistics

Results are expressed as mean ± SEM of at least three independent experiments, each performed in three to six replicates. Student’s t-test or when necessary analysis of variance (ANOVA) using XLSTAT were used to compare mean levels (*p<0.05).
